# Adaptive Kernel Convolutional Stereo Matching Recurrent Network

**DOI:** 10.3390/s24227386

**Published:** 2024-11-20

**Authors:** Jiamian Wang, Haijiang Sun, Ping Jia

**Affiliations:** 1 Changchun Institute of Optics, Fine Mechanics and Physics, Chinese Academy of Sciences, Changchun 130033, China; wangjiamian20@mails.ucas.ac.cn (J.W.); jiap@ciomp.ac.cn (P.J.); 2 University of Chinese Academy of Sciences, Beijing 100049, China

**Keywords:** stereo matching, GRU, adaptive, matching attention

## Abstract

For binocular stereo matching techniques, the most advanced method currently is using an iterative structure based on GRUs. Methods in this class have shown high performance on both high-resolution images and standard benchmarks. However, simply replacing cost aggregation with a GRU iterative method leads to the original cost volume for disparity calculation lacking non-local geometric and contextual information. Based on this, this paper proposes a new GRU iteration-based adaptive kernel convolution deep recurrent network architecture for stereo matching. This paper proposes a kernel convolution-based adaptive multi-scale pyramid pooling (KAP) module that fully considers the spatial correlation between pixels and adds new matching attention (MAR) to refine the matching cost volume before inputting it into the iterative network for iterative updates, enhancing the pixel-level representation ability of the image and improving the overall generalization ability of the network. At present, the AKC-Stereo network proposed in this paper has a higher improvement than the basic network. On the Sceneflow dataset, the EPE of AKC-Stereo reaches 0.45, which is 0.02 higher than the basic network. On the KITTI 2015 dataset, the AKC-Stereo network outperforms the base network by 5.6% on the D1-all metric.

## 1. Introduction

Binocular stereo matching has always been a focal point in research on stereo vision, emulating human binocular vision to recognize 3D information within the field of view [1]. The binocular camera takes two viewpoint images of the same scene and estimates the disparity by finding the horizontal correspondence between the corrected left and right images [2], and then obtains the depth map. Depth map has a wide range of applications. Because it can record the distance between objects in the scene and the camera, it has a wide range of applications in robot navigation [3,4], visual basic models [5], smart healthcare [6], 3D measurement [7], and augmented reality [8,9]. In recent years, with the support of large synthetic datasets [10,11,12], stereo matching methods based on Convolutional Neural Networks (CNNs) have elevated the accuracy of disparity estimation to a new level [13,14,15]. However, due to various real-world challenges, achieving both high accuracy and efficiency is crucial for practical applications.

Traditional stereo matching algorithms [16,17,18] typically involve four steps: computing the matching cost, cost aggregation, disparity computation, and disparity refinement [1]. And the matching process can be divided into two types: global matching methods and local matching methods. Since the high-efficiency local algorithms [19] are based on the assumption that the disparity of local windows is the same, which is not true in many cases, it will lead to poor matching performance. The global algorithms with better performance [20,21], although achieving better matching results through constraints such as smoothness constraints between two-dimensional adjacent pixel disparities, have a larger memory footprint and slower speed. In this case, in order to combine the advantages of the above two methods while avoiding their disadvantages, the semi-global matching method [22,23] was proposed. The disparity map obtained through the semi-global matching method was not significantly different in effect from the global algorithm, but there was a significant improvement in algorithm efficiency, making semi-global matching a widely used method at the time. However, in open-world environments, traditional methods like these still struggle with issues such as image noise, uneven lighting, and large textureless areas. It is therefore essential to develop targeted solutions to effectively handle real-world scenarios, particularly outdoor images.

In recent years, with the development of deep learning, each step in traditional stereo matching methods can be replaced by deep neural networks to improve performance [24]; thus, learning-based methods have been widely used in the field of binocular stereo matching. Compared to traditional methods, learning-based approaches produce more accurate and smoother disparity maps [14,15] and offer a slight advantage in computation speed [14,25]. The first end-to-end stereo matching network, DispNetC [11], computes a correlation volume from the features of the left and right images and uses a CNN to directly regress the disparity map. Since then, various learning-based methods have been proposed, and cost volume and correlation volume have been introduced into the network structure. GCNet [26] proposes using cascaded features to construct cost volumes and employing 3D convolution for cost aggregation. GA-Net [27] notes the drawbacks of 3D convolution and designs a semi-global aggregation layer and a local-guided aggregation layer, and tries to replace it with these two guided aggregation layers to further improve the accuracy. Meanwhile, GwcNet [28] proposes a group-wise correlation cost volume to enhance similarity measurements.

As mentioned above, most current algorithms use 2D CNNs to extract features, forming a cost volume that is subsequently fed into cost aggregation and regularization modules composed of 2D or 3D CNNs to compute the final disparity map. However, the aggregated information is often redundant [29], because it just concatenates all the information on a large number of feature dimensions. It does not require so much information to predict the disparity, and too much redundant information will have a bad impact on the network [30]. In fact, with the advancement of camera technology, the quality and resolution of initial images have greatly improved, leading to a significant amount of high-frequency information being disregarded during the aforementioned computation processes. This results in relatively blurry disparity maps lacking in detail, leading to insufficient matching accuracy and blurred depth estimation for the details. Consequently, the above algorithms are no longer compatible with the rapid advancements in current camera technology.

To adapt to higher-resolution images, the current state-of-the-art stereo methods [15,31,32,33] use an iterative structure based on ConvGRU [34] and have shown higher performance on both high-resolution images and standard benchmarks. Different from the above methods, the iterative approaches bypass the computationally expensive cost aggregation operations by only calculating the visual similarity between pixels at the same height. This substitutes the four-dimensional cost volume with a three-dimensional cost volume, while introducing multi-stage GRUs to progressively update the disparity map. This solution allows for the direct computation of cost volumes using high-resolution images, enhancing the ability of the update operators to propagate information across the image, and making it more suitable for high-resolution images. For instance, RAFT-Stereo [15] utilizes multi-layer convolutional gated recurrent units (ConvGRUs) [34] to cyclically update the disparity map using local cost volume retrieved from All-Pairs Correspondences (APCs); IGEV-Stereo [35] constructs a combined geometrically encoded volume to encode geometric and contextual information as well as local matching details, which is iteratively indexed to update disparity to make full use of all information. However, using iterative methods in place of cost aggregation results in the original cost volume lacking spatial-level information, and failing to better exploit the inherent geometric advantages present in stereo images. This leads to difficulties in handling pathological cases characterized by local blurriness, such as thin-structured objects (e.g., fences, wires) and textureless regions (e.g., highly reflective surfaces like glass). Another challenge is that due to their limited generalization ability, they are often less effective when applied to real-world scenarios than on specific datasets [36].

Therefore, the purpose of this article is to explore a better stereo matching method to make up for the shortcomings brought by the iterative method based on the GRU structure and solve the problem of disparity estimation in the above ill-posed regions. This article proposes a new design based on the IGEV-Stereo network, which can effectively improve the accuracy of the stereo matching network, extract features more accurately, make full use of the effective information of the images, and process thin structures and textureless areas more perfectly. The main contributions can be summarized as follows:

- In the feature extraction phase, this paper proposes the Kernel Normalization Convolution Layer-based Adaptive Spatial Pyramid Pooling (KAP) module to extract multi-scale features from images. This approach takes into full account the spatial correlation between pixels, thereby enhancing the performance of the deep network. The KAP module extracts more plentiful contextual features, enabling the network to better handle issues such as overexposure, thin structures, and low-texture regions, while also allowing the network to learn error correction capabilities.
- For the cost matching computation phase, this paper believes that conventional methods of connecting two cost volumes and then performing regularized correlation volume calculations struggle to fully exploit the advantages of the two image samples. To obtain a more reliable correlation volume, this paper proposes a new attention module (MAR) that integrates the regularized cost volume to produce a more accurate similarity measure. It not only effectively utilizes the effective geometric information of the binocular images, but also reduces the number of subsequent GRU iterations.
- Furthermore, this paper also utilizes a kernel convolution-based gated recurrent unit [37] as the iterative update operator within the main network framework, which retrieves features from the previously obtained stereo matching correlation volume and regressively updates the disparity. It enables GRUs to consider wider contextual information around each pixel, promotes the fusion of local and global information, and provides a more comprehensive understanding of the feature space.

**Figure 1:**
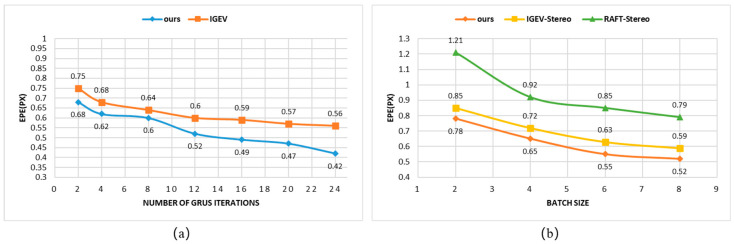
(**a**) shows the performance comparison between the AKC-Stereo network proposed in this article and the base network on the KITTI 2015 dataset with the change in the number of iterations; num_steps is set to 20,000, the abscissa is the number of iterations, and the ordinate is the EPE endpoint error data. In the figure, the orange part represents the performance of the base network, namely IGEV-Stereo, as the number of iterations changes, and the blue part represents the performance of the AKC-Stereo network as the number of iterations changes. (**b**) shows the effect of smaller batch size on the performance of the AKC-Stereo network proposed in this article (orange lines), RAFT-Stereo (green lines), and IGEV-Stereo (yellow lines) on the KITTI 2015 dataset with equal training rounds. The abscissa is the batch size number, and the ordinate is the EPE endpoint error data.

This article demonstrates the effectiveness of the proposed method on several publicly available datasets of binocular images. Due to the mature and widely used evaluation benchmarks of the KITTI dataset, the KITTI 2015 dataset is used as an example here. The AKC-Stereo network proposed in this article outperforms IGEV-Stereo [35] and CREStereo [33] by 5.6% and 11.24% on the D1-all metric in KITTI 2015 dataset [38], respectively. In the qualitative test, the AKC-Stereo in this article performs more excellently in light reflection regions and thin-structure regions, and also demonstrates better cross-dataset generalization ability. As shown in Figure 1, the accuracy of AKC-Stereo in this article is better than that of the basic network IGEV-Stereo. In Figure 1a, it can be seen that AKC- Stereo achieves a smaller EPE (that is, 0.52) using only 12 GRU iterations than IGEV-Stereo using 28 GRU iterations (that is, 0.56 EPE). In Figure 1b, it can be observed that AKC-Stereo also improves significantly for small batch training, which also helps the network to converge faster and reduce the footprint during training.

## 2. Materials and Methods

**Figure 2:**
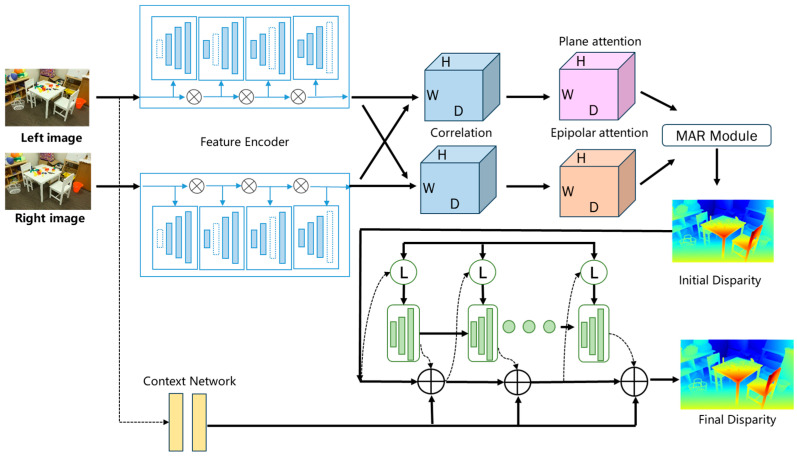
The overall structure diagram of the proposed AKC-Stereo. AKC-Stereo first constructs a multi-scale adaptive feature extractor (KAP), then computes the matching correlation volume by grouping correlation method, and then preliminarily refines it using the matching attention (MAR) module. Finally, the refined correlation volume is fused with the context-encoded features obtained through residual blocks, and the combined data are fed into the GRU iteration for further optimization through iterative updates.

In this section, the structure of AKC-Stereo is described in detail (as shown in Figure 2), which consists of an adaptive multi-scale feature extractor KAP based on kernel-normalized convolutional layers, a matching attention MAR module that combines disparity and epipolar self-attention information, a GRU update algorithm based on kernel convolution, and other basic modules.

### 2.1. Feature Extractor

The feature extractor consists of two parts: the context encoder, which extracts multi-scale contextual features, and the feature encoder applied to the left and right images.

The context encoder is based on IGEV-Stereo [35] and is composed of a series of residual blocks and downsampling layers. It generates multi-scale contextual features at 1/4, 1/8, and 1/16 resolutions of the input images, which have 128 channels. These contextual features are used to initialize the hidden state of the update operators and are injected into the GRU update operators during each iteration.

For the feature encoder, this paper was inspired by the success of the spatial pyramid pooling method in image segmentation technology [39]. By using dilated convolutions, multiple parallel convolutional layers with different dilation rates are employed to extract features, which are further processed and fused to produce the final result. This method allows the combination of semantic information with different receptive field sizes without sacrificing resolution (i.e., no downsampling), improving the precision of feature extraction. However, the superposition of dilated convolutions is not friendly to the disparity estimation task of dense pixels such as stereo matching, which will lose many pixel-level details of the object. To address this issue, this paper combines kernel convolution normalization and adaptive offset to create a variant of this approach, namely the KAP module, which will be described in detail in the following sections.

#### 2.1.1. Adaptive Offset

**Figure 3:**
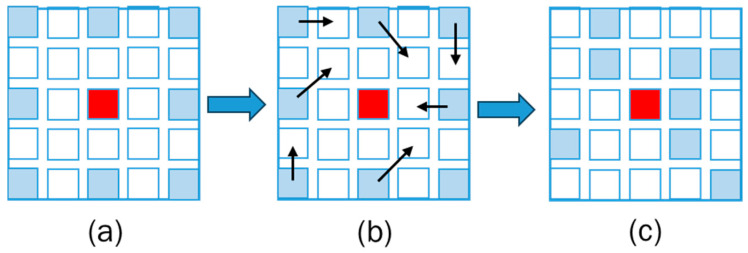
(**a**) shows the sampling search window with a normal dilation rate of 3 for feature extraction; (**b**) shows the positions where the sampling points should be shifted after adding offsets; (**c**) illustrates the more precise sampling search window obtained after the offsets are applied.

Aiming at the problem that the dilated convolution will lose the dense features of the object, in order to obtain dense global contextual information and establish dependency between pixels, this paper extends the deformable convolutions [40] to multi-scale pyramid pooling layers. By utilizing learnable offset to create adaptive search windows for multi-scale feature extraction, we achieved adaptive sampling of the input feature maps without increasing memory and computational costs. This enables the network to dynamically adjust the receptive field according to the shape and orientation of objects, thereby capturing more relevant and discriminative features. Figure 3 shows how offset changes the formation of feature extraction boxes.

In feature extraction, $h\left(p_{i}\right)$ represents the weight associated with each pixel $p_{i}$; the mapping relationship of any point $p_{0}$ in the feature map y relative to point $p_{i}$ in the original image x can be expressed by Equation (1):

(1) $$y(p_{0})=\sum_{p_{i}\in y}h(p_{i})\cdot x(p_{0}-p_{i})$$

After applying an offset Δp to the convolution, the mapping relationship is transformed into Equation (2):

(2) $$y(p_{0})=\sum_{p_{i}\in y}h(p_{i})\cdot x(-p_{i}+\Delta p)$$

**Figure 4:**
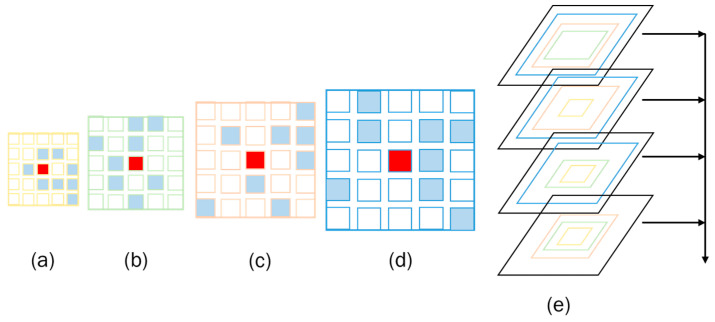
The figure illustrates some adaptive search windows with varying dilation rates for a 3 × 3 convolutional kernel. Specifically, (**a**) shows the adaptive search window with a dilation rate of 1; (**b**) displays the adaptive search window with a dilation rate of 3; (**c**) presents the adaptive search window with a dilation rate of 4; and (**e**) shows the fusion of features into four feature maps, respectively, by taking three layers of (**a**–**c**), three layers of (**b**–**d**), three layers of (**a**,**b**,**d**), and three layers of (**a**,**c**,**d**), and then the residual connection operation is performed to obtain the final feature map.

Firstly, this paper uses four parallel adaptive convolutional layers with different dilation rates (1, 3, 4, and 5) to generate more refined and comprehensive multi-scale features. Then, the extracted feature maps containing features of different scales are fused. In order to save computational cost and not lose information, only three of the feature maps obtained by convolution layers with different sampling rates were selected each time for fusion, that is, the feature maps obtained by adaptive convolution layers with expansion rates of 3, 4, and 5 are linked in block1. And they are normalized by kernel normalized convolution. In block 2, the feature maps obtained by linking adaptive convolutional layers with dilation rates of 1, 4, and 5 are also normalized using kernel normalization convolution. After that, the same process is applied to block3 and block4, using convolutional layers with dilation rates of (1, 3, 5) and (1, 3, 4), respectively. Finally, after normalization, we obtain a total of 4 blocks, which are eventually combined through residual cascading to produce the final feature map, as illustrated in Figure 4. Among them, the adaptive offset also undergoes end-to-end learning during the training process, ensuring that the network optimally adapts to the shape and scale of the target objects, thereby producing more accurate and detailed feature maps.

#### 2.1.2. Kernel Normalized Convolutional Feature Fusion

Through a large number of experiments, it is found that in the final multi-scale feature fusion of the feature extraction stage, the effectiveness of BatchNorm [41] will be greatly reduced when only small batch data can be used for input training if the environment memory is limited. In this regard, we refer to kernel-normalized convolution (KernelNorm) [37] instead of BatchNorm. Kernel normalization first applies random dropout [42] to the normalization units to obtain dropout units, and then normalizes the original normalization units by calculating the average value and variance of the dropout units. Its hyperparameters include kernel size k, stride s, padding d, and dropout probability p.

(3) $$U^{\prime }=D_{p}\left(U\right)$$

(4) $$\hat{U}=\frac{U-\mu_{u^{\prime }}}{\sqrt{\sigma_{u^{\prime }}^{2}+\epsilon }}$$

In Equations (3) and (4), $D_{p}$ represents the dropout operation; U stands for the old normalization unit; $U^{\prime }$ signifies the dropout units; $\mu_{u^{\prime }}$ and $\sigma_{u^{\prime }}^{2}$ correspond to the average value and variance of the dropout units; and $\hat{U}$ is the new normalization unit.

**Figure 5:**
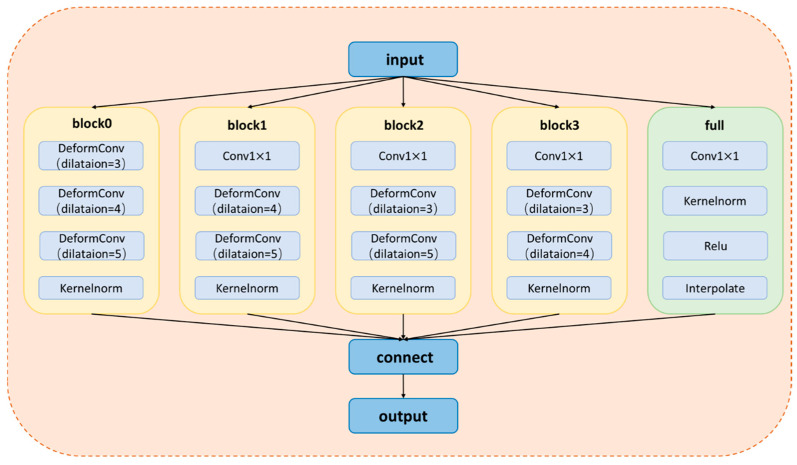
The concrete structure diagram of the KAP module.

Applying kernel normalization to the backend of each block ensures that the computation results do not depend on the statistical information of the entire batch. This approach emphasizes local information over global information within the feature space and reduces the dependency of multi-scale feature extraction on batch size, which is crucial for stereo matching tasks involving images under different scales and conditions. Moreover, by normalizing through kernel normalized convolution, the model is less prone to overfitting on specific scales or features, enhancing its generalization capabilities to unseen data. The specific network structure of the KAP module is illustrated in Figure 5.

### 2.2. Matching Attention

In the stage of calculating matching cost, there are three main steps: constructing the initial cost volume, generating matching attention weights, and feeding the final cost volume into the GRU-based update operator for further iterative refinement and optimization of the disparity map.

Usually, the construction of the initial cost volume is calculated using the group correlation method [28], that is, the channels of the left and right feature matrices are uniformly grouped along the channel dimension at a disparity level according to the vector inner product. Then, their inner product is calculated group by group, and the average value is calculated on the dimension of dim = 2 to obtain a three-dimensional matrix. When all disparity levels are traversed, the resulting matrices are split and concatenated to obtain the grouped correlation cost volume (group-corr volume).

If there are $N_{c}$ channels in common, dividing them into $N_{g}$ groups, the g-th feature groups $f_{l}^{g}$ and $f_{r}^{g}$ are composed of the $\left\{g\frac{N_{c}}{N_{g}},\;g\frac{N_{c}}{N_{g}}+1,\;\ldots ,\;g\frac{N_{c}}{N_{g}}+\left(\frac{N_{c}}{N_{g}}-1\right)\right\}$ channels of the original features $f_{l}$ and $f_{r}$, and the correlation of all feature groups g and all disparity levels d can be expressed by Equation (5).

(5) $$C_{cor}\left(d,x,y,g\right)=\frac{1}{N_{c}/N_{g}}\left\langle f_{l}^{g}\left(x,y\right),f_{r}^{g}\left(x-d,y\right)\right\rangle$$

**Figure 6:**
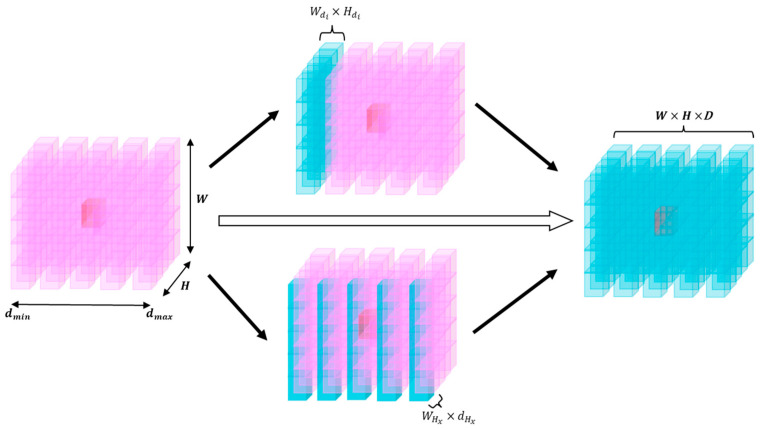
The pink cube on the left is the 3D correlation volume of size W×H×D calculated by correlation. The cubes in the middle, positioned above and below, represent the disparity-level attention calculated for the $W_{d_{i}}\times H_{d_{i}}$ plane and the epipolar-line-level attention calculated for the $W_{H_{x}}\times d_{H_{x}}$ plane, respectively. The blue cube on the right illustrates the refined correlation volume after being processed by the MAR matching attention module. This refined correlation volume is then fed into the GRU for iterative updates to further optimize the disparity map.

This paper believes that the traditional approach of connecting two correlation volumes and performing regularized correlation volume computation is insufficient to fully utilize the advantages of two image samples. To obtain a more reliable correlation volume, the initial correlation volume is filtered to emphasize useful information and suppress irrelevant information. This paper proposes a novel hybrid matching attention method to preliminarily refine the initial correlation volume. This method applies criss-cross attention [43] (as shown in Figure 6) to traverse the initial 3D correlation volume from both the disparity and epipolar-line directions, and performs preliminary optimization on it. This method reduces the number of iterations and improves accuracy for the next GRU iteration, enhancing pixel-level representation while reducing computational complexity and memory usage.

As shown in Figure 6, firstly for each disparity level $d_{i}$($i\in [d_{min},\;d_{max}]$), we extract the $W_{d_{i}}\times H_{d_{i}}$ plane and apply the criss-cross attention mechanism [43] to traverse it. For any spatial position $Q_{w_{n},h_{m}}$ within it, we calculate the correlation weights $A_{w_{n},h_{m}}$ for positions $Q_{w_{n},h}$ and $Q_{w,h_{m}}$, which align in a cross shape centered at $Q_{w_{n},h_{m}}$. Thus, $A_{w_{n},h_{m}}$ contains the mutual mapping information of $Q_{w_{n},h_{m}}$ corresponding to any point in this cross-position relationship. Then, the above operation is repeated once, ensuring that for any other spatial position $K_{w_{a},h_{b}}$, the correlation weights $A_{w,h}$ corresponding to $Q_{w_{n},h_{m}}$ can always be computed. At this point, after refining via attention, the plane obtains the disparity-level attention described by Equation (6).

(6) $$\Phi_{W_{d_{i}}\times H_{d_{i}}}^{\prime }=\sum_{w_{n},h_{m}\in W_{d_{i}},H_{d_{i}}}{(A}_{w,h}Q_{w_{n},h_{m}}+\Phi_{W_{d_{i}}\times H_{d_{i}}})$$

Then, the epipolar-level attention correlation weights $A_{w,d}$ are computed for any spatial position $Q_{w_{n},h_{m}}$. Considering that binocular images typically consist of left and right images, based on the epipolar constraints of epipolar geometry, the $W_{H_{x}}\times d_{H_{x}}$ plane within $H_{x}(x\in [H_{min},\;H_{max}]$) is extracted. By repeating the above operation, the epipolar-level attention Equation (7) can be obtained.

(7) $$\Psi_{W_{H_{x}}\times d_{H_{x}}}^{\prime }=\sum_{w_{n},d_{m}\in W_{H_{x}},d_{H_{x}}}{(A}_{w,d}Q_{w_{n},d_{m}}+\Psi_{W_{H_{x}}\times d_{H_{x}}})$$

Finally, by integrating the two aforementioned layers of attention mechanisms, the matching attention formula can be derived as Equation (8).

(8) $$\Omega_{H\times W\times D}=\sigma \left(\Psi_{W_{H_{x}}\times d_{H_{x}}}^{\prime }(\Phi_{W_{d_{i}}\times H_{d_{i}}}^{\prime })+\Phi_{W_{d_{i}}\times H_{d_{i}}}^{\prime }(\Psi_{W_{H_{x}}\times d_{H_{x}}}^{\prime })\right)$$

Hence, the final correlation can be expressed as Equation (9).

(9) $$C_{mar}\left(d,x,y,g\right)=\frac{1}{N_{c}/N_{g}}\left\langle f_{l}^{g}\left(x,y\right)\cdot \Omega_{H\times W\times D},f_{r}^{g}\left(x-d,y\right)\cdot \Omega_{H\times W\times D}\right\rangle$$

Through the matching attention mechanism that combines the self-attention information of both parallax and epipolar directions, the correlation volume is preliminarily refined, and the result is input into the GRU-based update operator for further iterative disparity map optimization, which can make the network more effectively learn different features of the input data while taking into account the interdependence between the channel and the global information. It not only effectively utilizes the effective geometric information of the binocular images and improves the generalization ability of the network, but also greatly reduces the difficulty of the network to find the correct matching points in the search space.

### 2.3. Kernel Convolution-Based GRU Iterative Update Operator

According to the baseline network, the disparity update uses a sequence of gated recurrent units (GRUs) to combine all the previously acquired data. Starting from an initial starting point $d_{0}$ = 0, the update operator predicts a sequence of disparity fields {$d_{1}$, …, $d_{N}$}. Each iteration, it produces an update direction Δd, which is applied to the current estimate: $d_{k+1}=\Delta d+d_{k+1}$. The update operator takes disparity, correlation, and potential hidden states as input and outputs an update Δd and the updated hidden state. Mimicking the steps of the optimization algorithm, each iteration uses the current disparity estimate to index the correlation volume, producing a set of correlation features, and then the GRU updates the hidden state, and the new hidden state is then used to predict the disparity update.

The traditional GRU is well suited for handling sequential data but has certain limitations in directly capturing spatial dependencies. To address this, this paper adopts a GRU based on kernel convolution [37] as the iterative update operator within the main network framework. The GRU retrieves features from the stereo matching cost volume obtained earlier, and regresses iterative disparity updates.

First, the soft argmin function is used to regress the matching correlation volume to the initial disparity. The disparity is then updated through three GRU iterative update layers, each containing three kernel convolution layers. The activation function used is the sigmoid function, which connects and injects correlation, disparity, and contextual features into the GRU. The hidden state is then updated, and the new hidden state is used to predict the disparity update. During training, all stages of disparity iterative updates share the same weights, eliminating the need for fine-tuning.

The input-to-hidden and hidden-to-hidden transformations in the GRU make use of kernel convolutions, ensuring that spatial information is preserved and propagated efficiently through recurrent connections. By integrating kernel convolutions into the GRU, the network can process both spatial and temporal information, enabling the GRU to consider a wider range of contextual information around each pixel, facilitating the fusion of local and global information and providing a more comprehensive understanding of the feature space, especially in challenging regions such as thin structures and highly reflective regions. It enhances the network’s ability to calculate the scene structure of larger areas.

### 2.4. Loss Function

Since smooth loss is considered robust to disparity discontinuities and outliers, we use smooth loss as our training loss function. The smooth L1 loss is computed between the predicted values and ground truth over the entire prediction sequence, using an exponentially weighted L1 distance similar to IGEV [32] as the loss function. For all predicted disparities $d_{i}\left\{d_{1},\ldots ,d_{N}\right\}$, given ground truth disparities $d_{gt}$, the loss function L is defined as Equations (10) and (11).

(10) $$L_{\mathrm{cor}\;}=Smooth_{L_{1}}\left(d_{0}-d_{gt}\right)$$

(11) $$L=L_{\mathrm{cor}\;}+\sum_{i=1}^{N}\gamma^{N-i}\left.|d_{i}-d_{gt}\right.{|}_{1},\;\gamma =0.9$$

## 3. Results

### 3.1. Implementation Details and Evaluation Metrics

In this experiment, the system used is Ubuntu 20.04 with an NVIDIA RTX 3090 GPU, 32 GB of memory, CUDA 11.2, and Python 3.9. Images are randomly cropped to 320 × 736 and trained using the same data augmentation methods as in [15]. During training, the network was trained for 20 k steps with a batch size of 2, using a single-cycle learning rate schedule with a learning rate of 0.0002, and 16 update iterations were employed.

Generally, the performance evaluation metrics used in stereo matching tasks are EPE, D1, and D1-BG [44]. In addition, we added the size of the model parameters as well as the computation time to the evaluation metrics, counting convolutional layers, fully connected layers, and other layers with parameters in the network structure.

EPE refers to the pixel-level average Euclidean distance between the predicted depth map and the true depth map, which reflects the disparity estimation error information of all pixels of the whole image. It is calculated as shown in Equation (12).

(12) $$EPE=\sqrt{\left\{{\left(x_{\left\{pred\right\}}-x_{\left\{true\right\}}\right)}^{2}+{\left(y_{\left\{pred\right\}}-y_{\left\{true\right\}}\right)}^{2}\right\}}$$

Both D1 and D1-BG are the percentage of erroneous pixels over all pixels. Generally, when the absolute disparity error of a pixel is greater than three pixels, the pixel is labeled as an error pixel. This index reflects the proportion of erroneous pixels in the whole image [45]. D1 is the proportion of incorrectly predicted pixels in the whole region and D1-BG is the proportion of incorrectly predicted pixels only in the background region; these are calculated by Equations (13) and (14), respectively.

(13) $$D1=\frac{1}{N}\;\sum_{\left\{i=1\right\}}^{\left\{N\right\}}\{\left|D_{\left\{pred\right\}\left(i\right)}-D_{\left\{true\right\}\left(i\right)}\right|>3\}$$

(14) $$D1-BG=\frac{1}{N}\sum_{\left\{i=1\right\}}^{\left\{N\right\}}\left\{\left|D_{\left\{pred-bg\right\}\left(i\right)}-D_{\left\{true-bg\right\}\left(i\right)}\right|>3\right\}$$

### 3.2. Datasets

This paper evaluates the proposed AKC-Stereo on several public datasets, including Sceneflow [2], ETH3D [46], Middlebury [47], KITTI-2012 [48], and KITTI-2015 [38]. Compared with the basic IGEV-Stereo method [32], AKC-Stereo improves the accuracy and also makes the network perform better in highly reflective areas and thin-structure areas.

Sceneflow [2] is a large-scale synthetic dataset that contains comprehensive data of more than 35,000 stereo image pairs, and contains the real values of disparity, optical flow, and scene flow. It is suitable for training convolutional networks and can also be used for the evaluation of other methods, and the image size is 540 × 960.

KITTI 2012 [48] and KITTI 2015 [38] are two datasets widely used in the field of autonomous driving, composed of data from real driving scenes. They provide sparse ground truth disparity values obtained using LiDAR, for the evaluation of tasks such as stereo matching and optical flow. The KITTI 2012 dataset contains 194 training image pairs and 195 testing image pairs, with an image resolution of 1226 × 370. Compared to the 2012 version, the KITTI 2015 dataset includes more dynamic scenes and consists of 200 training image pairs and 200 testing image pairs.

Middlebury 2014 [47] is an indoor dataset that offers 23 pairs of high-resolution images captured under different lighting conditions.

ETH3D [46] is a grayscale dataset consisting of 27 pairs of monochrome stereo images. It provides disparity samples obtained by laser scanning, covering both indoor and outdoor scenes.

### 3.3. Ablation Experiment

Ablation studies play a crucial role in interpreting the effectiveness of different components and strategies in the ever-changing deep stereo matching field [49]. In order to verify the effectiveness of integrating the matching attention mechanism and the adaptive multi-scale feature extraction module into the proposed network, this paper designs ablation experiments on the KITTI 2015 dataset. For all models in these experiments, it is set to 16 times in the number of iterative update operators. Using IGEV-Stereo as the backbone, this paper separately incorporated the adaptive multi-scale feature extraction module (KAP) and the matching attention mechanism (MAR) to validate their effects on improving the model’s performance. As shown in Table 1, both the proposed adaptive spatial pyramid pooling layer (KAP) and the matching attention mechanism (MAR) significantly enhance prediction accuracy. By fully considering the spatial correlation between pixels and the geometric relationships in stereo images, the initial disparity fed into the iterative update network becomes more precise and substantially reduces prediction error.

### 3.4. Benchmark Evaluation

The proposed AKC-Stereo is compared with the state-of-the-art stereo matching methods published by KITTI 2012 and 2015. On the Sceneflow dataset, the EPE of the AKC-Stereo reaches 0.45, which is more accurate than most recent networks, and 0.02 higher than IGEV-Stereo. Detailed quantitative comparisons are provided in Table 2.

**Figure 7:**
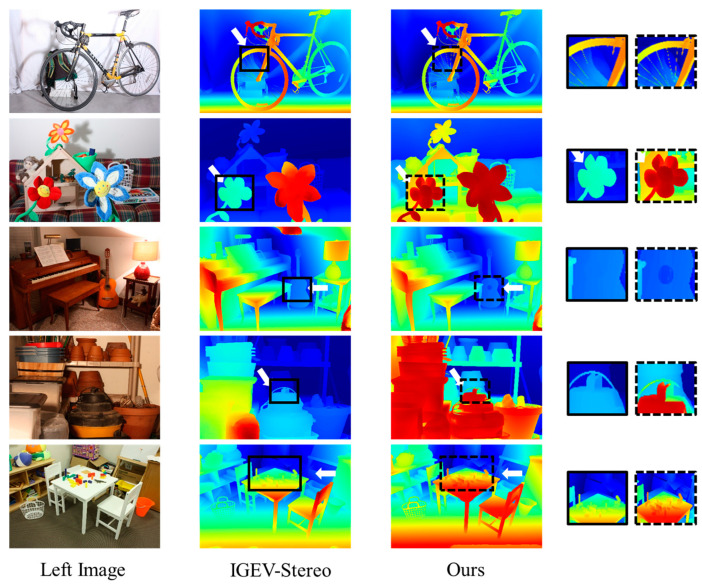
Qualitative results for Middlebury. The first column shows the original images (left images) in the dataset, and the second and third columns show the results of IGEV-Stereo and the AKC-Stereo network proposed in this article, respectively. The proposed network exhibits better results for detailed parts as well as regions with a background.

Figure 7 shows the visual comparison between the computational results of the AKC-Stereo network proposed in this paper and IGEV-Stereo on the Middlebury dataset, where the proposed method is more robust to detailed regions and regions with a background.

In this paper, we conducted a quantitative evaluation of our network on the KITTI 2012 and KITTI 2015 datasets, as shown in Table 3. Compared to most state-of-the-art algorithms in recent years, the AKC-Stereo network proposed in this paper achieved superior performance on many metrics for both the KITTI 2012 and 2015 datasets. For the KITTI 2012 dataset, for the 3-all error metric, the proposed network outperformed IGEV-Stereo and CREStereo by 9.59% and 8.33%, respectively. In the KITTI 2015 comparison, the proposed network exceeded IGEV-Stereo and CREStereo by 5.6% and 11.24%, respectively, for the D1-all metric. It is evident that the proposed algorithm has shown significant improvements after optimization. However, it is slightly deficient in speed, which is exactly the area we will continue to improve in the next step.

**Figure 8:**
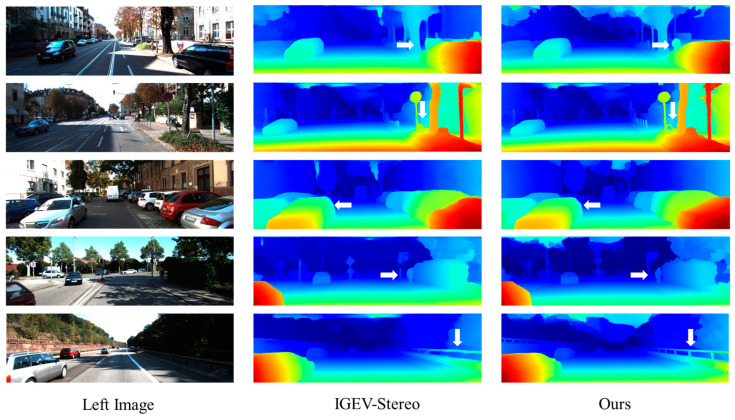
Qualitative results on the KITTI2015 dataset. The first column shows the left image of the original image in the dataset, the second column shows the operation results of the baseline IGEV-Stereo, and the third column shows the operation results of the AKC-Stereo network. From the direction indicated by the arrow, it can be seen that the AKC-Stereo network proposed in this article performs exceptionally well in areas with high light reflection such as signs and areas with thin structures such as railing.

Figure 8 shows the qualitative comparison results of the proposed network in the 2015 KITTI dataset, both after 50 k steps of training under the condition of batch size 2. It can be seen that the AKC-Stereo network performs very well in regions with light reflection regions and thin structures, with sharper contours and edges. 

### 3.5. Generalization Experiment

Since the real world is never a perfect dataset with balanced class distribution, no noise, no outliers, and uniform data distribution, the ability to generalize is therefore essential for a network to be able to adapt to new and unknown situations, rather than just performing well on the training data. The strength of the generalization ability directly reflects whether the patterns learned by the model are universal and applicable to a broader range of situations.

In this section, the generalization ability of the AKC-Stereo network proposed in this paper for unknown scenarios will be studied. The Sceneflow dataset is large and contains images under a variety of scenes and lighting conditions, while the Middlebury and ETH3D datasets are synthetic datasets and real scene datasets, respectively, which can more widely reflect complex situations in the real world. At present, most networks choose to use the Middlebury and ETH3D datasets for generalization ability testing. Therefore, in order to better compare with other advanced network models, the AKC-Stereo network model is also trained on Sceneflow, and directly evaluated on the training sets of Middlebury2014 and ETH3D. The quantitative results are shown in Table 4, and it can be seen that the proposed network achieves better results with more stable performance under the same settings.

## 4. Discussion and Conclusions

This paper proposes a novel adaptive kernel convolution stereo matching recurrent network for stereo matching. This network incorporates an adaptive multi-scale feature extraction module (KAP) and a new matching attention module (MAR) to perform multi-scale feature extraction and obtain a more reliable correlation volume. Through the KAP module, the learnable offset is used to form an adaptive search window to extract multi-scale image features, and the multi-scale features are fused by kernel convolution normalization. Through the new matching attention MAR module, the initial correlation volume is initially refined by self-attention in the two directions of disparity and epipolar line, which provides a more accurate basis for the subsequent GRU iterative update to calculate disparity.

The accuracy of the improved network AKC-Stereo is better than that of the basic network IGEV-Stereo. It outperforms most advanced algorithms developed in recent years across various metrics, particularly excelling in handling pathological regions in images, such as highly reflective surfaces and thin structures. Although the method proposed in this paper has some shortcomings in calculation speed, this is precisely the direction of further research and improvement in the next step.

## Figures and Tables

**Table 1:** The table presents the results of the ablation study of the proposed AKC-Stereo on the KITTI2015 dataset. KAP denotes the adaptive multi-scale feature extraction module, and MAR refers to the matching attention mechanism. The baseline is IGEV-Stereo, and num_steps is set to 20,000.

Model	EPE (px)	D1-All (%)	Time (s)	Params (M)
Baseline	0.57	1.90	**0.34**	**12.60**
KAP	0.50	1.62	0.45	13.22
MAR	0.47	1.64	0.48	13.21
**Full Model**	**0.42**	**1.52**	0.52	13.60

**Table 2:** Quantitative evaluation on Sceneflow dataset. Bold: best.

Model	PSMNet [50]	GwcNet [28]	AANet [25]	LEAStereo [13]	ACVNet [29]	IGEV-Stereo [35]	Ours
**EPE** **(px)**	1.09	0.76	0.87	0.78	0.48	0.47	**0.45**

**Table 3:** Quantitative evaluation on KITTI 2012 and KITTI 2015. The AKC-Stereo network proposed in this paper runs 16 updates at inference. Bold: best.

	KITTI 2012				KITTI 2015			Runtime (s)
Model	3-noc (%)	3-all (%)	EPE (px) noc	EPE (px) all	D1-bg (%)	D1-fg (%)	D1-all (%)	
PSMNet [50]	1.49	1.89	0.5	0.6	1.86	4.62	2.32	0.41
GwcNet [28]	1.32	1.70	0.5	0.5	1.74	3.93	2.11	0.32
CFNet [51]	1.23	1.58	**0.4**	0.5	1.54	3.56	1.88	0.18
ACVNet [29]	1.13	1.47	**0.4**	0.5	1.37	3.07	1.65	0.20
LEAStereo [13]	1.13	1.45	0.5	0.5	1.40	2.91	1.65	0.30
NMRF [52]	**1.01**	1.35	**0.4**	**0.4**	1.28	3.13	1.59	**0.09**
DMIO [53]	1.14	1.48	**0.4**	**0.4**	1.45	2.61	1.64	—
CREStereo [33]	1.14	1.46	**0.4**	0.5	1.45	2.86	1.69	0.41
RAFT-Stereo [15]	1.30	1.66	**0.4**	0.5	1.58	3.05	1.82	0.38
IGEV-Stereo [35]	1.12	1.44	**0.4**	**0.4**	1.38	2.67	1.59	0.18
**O** **urs**	1.03	**1.32**	**0.4**	**0.4**	**1.23**	**2.48**	**1.50**	0.52

**Table 4:** Generalization experiments. All models are trained on Sceneflow. The 2-pixel error rate is used for Middlebury 2014, and the 1-pixel error rate for ETH3D.

Model	Middlebury2014		ETH3D
	Half	Quater	
PSM [50]	25.8	14.2	23.8
GwcNet [28]	18.1	—	9.0
DSMNet [54]	13.8	8.1	6.2
RAFT-Stereo [15]	8.7	7.3	**3.2**
IGEV-Stereo [35]	7.1	6.2	3.6
**Ours**	**6.4**	**5.8**	3.3

## Data Availability

Data are contained within the article. The data presented in this study are available on request from the corresponding author. The data are not publicly available due to restrictions of privacy.
